# A New High-Performance Gadonanotube-Polymer Hybrid Material for Stem Cell Labeling and Tracking by MRI

**DOI:** 10.1155/2018/2853736

**Published:** 2018-07-10

**Authors:** Sakineh E. Moghaddam, Mayra Hernández-Rivera, Nicholas G. Zaibaq, Afis Ajala, Maria da Graça Cabreira-Hansen, Saghar Mowlazadeh-Haghighi, James T. Willerson, Emerson C. Perin, Raja Muthupillai, Lon J. Wilson

**Affiliations:** ^1^Department of Chemistry, The Smalley-Curl Institute, Rice University, M.S. 60, P.O. Box 1892, Houston, TX 77251-1892, USA; ^2^Stem Cell Center, Texas Heart Institute, Baylor St. Luke's Medical Center, MC 2-255, P.O. Box 20345, Houston, TX 77225-0345, USA; ^3^Department of Radiology, Baylor St. Luke's Medical Center, 6720 Bertner Avenue, MC 2-270, Houston, TX 77030-2697, USA; ^4^Department of Physics, University of Houston, Houston, TX 77004, USA

## Abstract

A gentle, rapid method has been developed to introduce a polyacrylic acid (PAA) polymer coating on the surface of gadonanotubes (GNTs) which significantly increases their dispersibility in water without the need of a surfactant. As a result, the polymer, with its many carboxylic acid groups, coats the surface of the GNTs to form a new GNT-polymer hybrid material (PAA-GNT) which can be highly dispersed in water (ca. 20 mg·mL^−1^) at physiological pH. When dispersed in water, the new PAA-GNT material is a powerful MRI contrast agent with an extremely short water proton spin-lattice relaxation time (*T*_1_) which results in a *T*_1_-weighted relaxivity of 150 mM^−1^·s^−1^ per Gd^3+^ ion at 1.5 T. Furthermore, the PAA-GNTs have been used to safely label porcine bone-marrow-derived mesenchymal stem cells for magnetic resonance imaging. The labeled cells display excellent image contrast in phantom imaging experiments, and transmission electron microscopy images of the labeled cells reveal the presence of highly dispersed PAA-GNTs within the cytoplasm with 10^14^ Gd^3+^ ions per cell.

## 1. Introduction

Among the many nanoparticles currently under investigation, carbon nanotubes (CNTs) have been and continue to be a key material because of their unique properties and extreme diversity of potential applications [1–5]. However, poor solubility of CNTs in water or organic solvents is a challenging limitation for many practical applications of this unique material [6]. To integrate CNT technology with medical and biological milieu, CNT solubility or suspendability needs to be improved for aqueous and biological media [7–9]. Toward this end, the two main approaches that have been used to increase dispersion of CNTs are (1) noncovalent functionalization of CNTs with surfactants, nucleic acids, peptides, polymers, or oligomers [10–14] and (2) CNT covalent functionalization [15–17].

Nanomaterials, including CNTs, have been extensively studied as diagnostic agents, for example, as contrast agents (CAs) for magnetic resonance imaging (MRI). MRI has become a standard clinical tool to obtain human anatomical and functional information noninvasively [18–20]. In current clinical approaches, CAs are sometimes administered to enhance signal intensity of MR images [21–23]. Because Gd^3+^ has seven unpaired electrons, giving it a high magnetic moment and relatively long magnetic relaxation time, most of these CA materials are small-molecule Gd^3+^-chelate compounds that disseminate uniformly throughout the vasculature [21, 22, 24, 25]. The efficacy of these MRI CAs is characterized by their relaxivity (*r*_1_), which is the water proton relaxation rate constant normalized to the concentration of the CA. These image-brightening agents shorten the water proton spin-lattice relaxation time (*T*_1_) by the magnetic coupling of their paramagnetic centers to the surrounding water proton nuclear spin [21, 22, 24]. Although most current clinically used Gd^3+^ ion-based CAs have acceptable safety profiles, they also possess very low relaxivities of only ∼4-5 at 1.5 T and 37°C [26–28].

In 2005, gadonanotubes (GNTs) were first reported, revealing a new strategy for sequestering Gd^3+^ ions by encapsulating them within (or upon) ultrashort carbon nanotube capsules (US-tubes) [29]. This new carbon nanostructure with a relaxivity as high as 160 mM^−1^·s^−1^ per Gd^3+^ ion at 1.5 T and 37°C is the highest-performing Gd^3+^-based MRI CA material discovered to date [30]. US-tubes are produced by first cutting full-length single-walled carbon nanotubes (SWCNTs, > 1 *µ*m in length) via a previously reported fluorination/pyrolysis method [31]. Thereafter, US-tubes (average length of 50 nm) are purified by treatment with HCl, debundled under Birch reduction conditions, and then mildly oxidized by HNO_3_ to produce carboxylic acid groups at US-tube defect sites. Mild sonication of US-tubes in the presence of Gd^3+^ ions in aqueous solution is then the final step in the preparation of the GNTs. To increase the dispersibility of the GNTs in biological media for *in vivo* studies, they have often been suspended in aqueous solution using Pluronic®-108 [32, 33], a nonionic and biocompatible surfactant. In further efforts to boost the dispersibility of GNTs in biological media, we recently produced a second-generation GNT material (PCP-GNTs) by covalently attaching benzoic acid groups via diazonium-based chemistry [34].

In the current study, we have produced yet a third-generation GNT material by coating the GNT outer surface using a mild in situ polyacrylic acid (PAA) polymerization procedure to produce a new highly water-dispersible PAA-GNT material. The motivation for producing this new material was twofold. First, surfactant-wrapped GNT materials are known to quickly shed the surfactant wrapping *in vivo* [2, 35–37], and it was hoped that the new PAA-GNT material would resist this process through stronger intermolecular attraction gained from using the GNTs as a scaffold for polymerization. By making GNTs water-soluble and stable in biological media, the potential to move this material toward vascular MRI applications for the first time is significantly greater. Secondly, the second-generation GNT material, with covalently attached benzoic acid groups (PCP-GNTs), is labor-intensive and time-consuming [34], and it was hoped that the new PAA-GNT material could be produced more quickly and using a much simpler synthetic process which has now been verified by the current study. Finally, we have also evaluated MRI performance of the new PAA-GNT material and employed the material to safely label and image porcine bone-marrow-derived mesenchymal stem cells (MSCs) as a demonstration of a valuable application for the material.

## 2. Experimental

### 2.1. Preparation of the PAA-GNTs

We followed the methods of Gizzatov et al., and US-tubes were prepared by a previously reported method [31, 34]. Briefly, 200 mg of SWCNTs (Carbon-Arc SWCNTs from Carbon Solutions Inc.) was fluorinated using 2% F_2_ in a He gas mixture with a flow rate adjusted to 15 cm^3^·min^−1^ along with H_2_ gas at a flow rate of 10 cm^3^·min^−1^ at 125°C for 2.5 h. The fluorinated product was then heated at 1000°C for 3 h under a continuous flow of Ar. The as-produced US-tubes were then sonicated in 200 mL of concentrated HCl for 60 min to remove metal impurities, washed with DI H_2_O, dried, and individualized by sonication for 60 min in 200 mL of dry THF and Na^0^ of equal weight to the US-tube sample. Next, US-tubes were refluxed in 200 mL of 6 M HNO_3_ for 15 min, washed with DI H_2_O, and dried. Loading of the US-tubes with GdCl_3_ was achieved by 1 h sonication in a 1 mM aqueous solution of GdCl_3_ to produce Gd@US-tubes or gadonanotubes (GNTs). The GNT product was washed with DI H_2_O until Gd^3+^ ions could not be detected in the filtrate (as determined by inductively coupled plasma optical emission spectrometry, or ICP-OES). GNTs were then further functionalized using an in situ polymer growth procedure. Briefly, 100 mg GNTs in 40 mL H_2_O was added to a 100 mL 3-neck flask. The mixture was sonicated for 30 min at 12 W and 55 kHz, and the well-suspended mixture was stirred vigorously at 50°C under N_2_ gas. Next, a solution of 1.5 mg potassium persulfate (KSP) dissolved in 200 mg acrylic acid was added with a tube pump at 5 mL·h^−1^, and the mixture was then let stir for 3 h. After completion of the reaction, the suspension was filtered through a 0.2 *µ*m PTFE membrane and the collected powder was redispersed in water with stirring for one day before being filtered. Finally, the resultant powder was dried in a vacuum oven at 80°C overnight to obtain the PAA-GNT sample. The maximum suspendability of the PAA-GNTs in water was determined to be ca. 20 mg·mL^−1^ by lyophilizing an aliquot of a supersaturated solution in which an excess of the PAA-GNTs was dispersed in 2 mL of H_2_O and left undisturbed for 24 h, after which 400 *µ*L of the supernatant solution was dried and the dry product (powder) was weighted using a microbalance. The obtained PAA-GNT material was then characterized with high-resolution transmission electron microscopy (HRTEM, JEOL 2100), scanning electron microscope equipped with energy dispersive spectrometry (EDS, FEI Quanta 400F), ICP-OES using a Perkin-Elmer Optima 8300 instrument, Raman spectroscopy using a ReNishaw inVia Raman microscope, and thermogravimetric analysis (TGA) using a Q-600 simultaneous TGA/DSC from TA Instruments. *In vitro* work was performed using PAA-GNTs which contained ca. 4.5% Gd by weight as determined by ICP-OES.

### 2.2. MR Imaging and Relaxometric Analysis

Phantom MR images of the PAA-GNT CAs were prepared by taking a 0.9 mg mL^−1^ aqueous dispersion of each sample. *T*_1_-weighted MR images of the samples were then determined at room temperature (RT) (25°C) using a commercial 1.5 T MRI scanner (Achieva, Philips Medical System, the Netherlands). A Q-body coil and an 8-channel wrist coil were used for radio-frequency transmission and signal reception, respectively. An inversion recovery prepared turbospin sequence was used to measure the *T*_1_ relaxation times of the samples (TR = 10000 ms; TE = 8 ms). The images were acquired over a field of view of 81 × 121 mm, with an acquired voxel resolution of 0.59 × 0.77 × 5.00 mm and a reconstruction matrix resolution of 0.24 × 0.24 × 5.00 mm. Following the inversion preparation, data acquisition commenced after inversion delay times (TI) of 200, 400, 800, 1200, and 1500 ms, and the *T*_1_ values were calculated using the standard inversion recovery equation. HPLC-grade water was used as a diamagnetic control. The samples were then digested in 26% HClO_3_ and reconstituted in 10 mL of trace metal-grade 2% HNO_3_ (aq) for determination of Gd^3+^ ion concentration by ICP-OES.

### 2.3. Stem Cell Labeling Experiments

The PAA-GNTs were used to intracellularly label porcine bone-marrow-derived mesenchymal stem cells (MSCs) harvested from three different animals. To prepare the stock labeling solution, the PAA-GNTs were suspended in water (100 *µ*M Gd^3+^, by ICP-OES) and the suspension was sterilized by UV light exposure for 3 h with rocking, which has been shown to be a procedure that does not cause damage to CNT materials [38]. MSCs were grown in T-175 flasks with alpha minimal essential medium (*α*MEM) containing 10% fetal bovine serum (FBS) and incubated at 37°C (95% relative humidity in 5% CO_2_ in air). Cells were expanded until the third passage prior to labeling. The PAA-GNT-labeled MSCs were prepared by adding the stock labeling solution directly to the *α*MEM (final concentration 20 *µ*M Gd^3+^) followed by incubation of the cells for 24 h with the CA. After collecting the cells, the suspension was passed through a 70 *µ*m nylon filter to eliminate cell aggregates and the cells were resuspended in 20 mL of *α*MEM. A known density gradient separation method was applied using a 50 mL conical tube to isolate the cells from excess of PAA-GNTs in solution, as well as from “heavy” cells which are labeled cells with PAA-GNTs on their cellular membrane [2]. To accomplish this, 10 mL of Ficoll-Paque (20°C, Sigma-Aldrich) was added to the bottom of the conical tube containing 20 mL of the cell suspension and the sample was centrifuged at 400 g for 20 min. The labeled MSCs were then isolated from the interface of the *α*MEM and the Ficoll-Paque using a plastic transfer pipette. Cells were then resuspended in fresh *α*MEM and centrifuged at 1500 rpm for 10 min to wash the residue of Ficoll-Paque in cells. Cell counts were obtained using a Beckman Counter MultiSizer 3. Unlabeled MSCs were used as control cells. Aliquots of labeled and unlabeled cell suspensions were collected and analyzed by ICP-OES (Perkin-Elmer Optima 8300 instrumentation) to determine the Gd^3+^ ion concentration in the cells. To prepare the samples, cells were heated and treated with two alternating additions of 500 *µ*L 70% trace metal-grade HNO_3_ and 26% HClO_3_, allowing the samples to dry between additions. Finally, the samples were diluted to 10 mL with an aqueous solution of 2% trace metal-grade HNO_3_ and 2% ethanol and finally filtered through a 0.22 *µ*m pore size syringe filter.

### 2.4. Viability of the PAA-GNT-Labeled MSCs

Fluorescence-activated cell sorting (FACS) was performed using a BD Biosciences LSRII Analyzer in order to determine the viability of the MSCs after being exposed to PAA-GNTs for 24 h. The experiment was run in triplicate using three different animal cell lines. A LIVE/DEAD viability/cytotoxicity assay kit (Life Technologies) was used to stain the cells: green-fluorescent calcein-AM indicates intracellular esterase activity in viable cells, while red-fluorescent ethidium homodimer-1 indicates dead cells when the cell membrane was compromised. Unlabeled MSCs were used as the positive control while unlabeled MSCs incubated with 70% methanol for 15 min were used as the negative control (dead cells). The dyes were added, and the samples were incubated in the dark at room temperature for 20 min prior to analysis.

### 2.5. MR Imaging of the PAA-GNT-Labeled MSCs

PAA-GNT-labeled MSCs were prepared as described above. Samples of 30 million unlabeled and labeled MSCs were separately centrifuged in a 1.5 mL Eppendorf tube to form cell pellets. The supernatant was carefully removed without disturbing the cell pellet. Cautiously, 500 *µ*L of 0.5% agar was added on top of cell pellet. The *T*_1_-weighted MR images of the labeled and unlabeled MSCs were acquired at RT using a commercial 1.5 T MRI scanner (Achieva, Philips Medical System, the Netherlands) with an inversion recovery prepared spin echo sequence (acquisition voxel size: 1.1 × 1.1 × 5 mm^3^; TR/TE: 6000 ms/11 ms). The experiment was repeated at various inversion times (TIs): 50, 100, 200, 400, 600, 800, 1200, 2000, 3000, and 4000 ms.

### 2.6. Transmission Electron Microscopy (TEM) Imaging of the PAA-GNT-Labeled MSCs

TEM analysis was performed to determine the subcellular localization of the PAA-GNT CAs. Labeled MSCs and unlabeled MSCs were centrifuged separately at 1500 rpm for 10 min to form a cell pellet. Without disturbing the pellet, the supernatant was removed, 3% glutaraldehyde was added, and the samples were left undisturbed for 2 days. Later, the samples were washed with 1X phosphate-buffered saline (PBS) and postfixed with 1% OsO_4_ for 1 h and then washed and dehydrated with increasing concentration of ethanol, and infiltrated with acetone and Epon 812 resin. Finally, the samples were embedded with 100% Epon 812 in a mold, cut into 1 mm sections, and stained with 1% methylene blue and 1% basic fuchsin. Ultrathin sections of 80 nm were cut from the sample block using a Leica EM UC7 ultramicrotome and framed on 100-mesh copper grids. Grids were stained with 2% alcoholic uranyl acetate and Reynold's lead citrate. The grids were examined using a JEOL 1230 TEM instrument equipped with an AMTV 600 digital imaging system at the Texas Heart Institute (Houston, TX).

## 3. Results and Discussion

### 3.1. Characterization of PAA-GNTs

**Figure 1:**
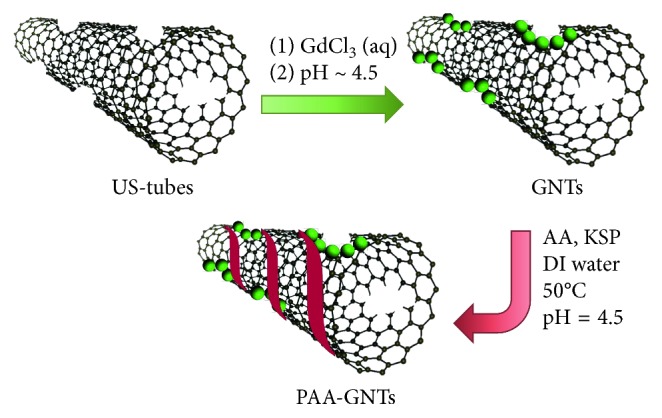
Schematic representation for the preparation of the PAA-GNTs.

GNTs were prepared as previously reported [29]. The concentration of the Gd^3+^ ions of the GNTs was determined to be 4.5 wt.% by ICP-OES. The surface of the GNTs was then functionalized via an in situ free radical polymerization of acrylic acid (AA), using potassium persulfate (KSP) as an initiator to prepare the resultant PAA-GNTs (Figure 1). To obtain the greatest dispersibility for the PAA-GNTs, control reactions were performed using empty US-tubes under different reaction conditions.

Dispersibility testing revealed that, by increasing the concentration of AA monomer to 0.17 M, the dispersibility of the PAA-GNT product was increased up to 40 times compared to untreated US-tubes. However, a further increase in the concentration of AA led to a higher degree of polymerization, which, in turn, reduced the suspendability of the PAA-GNT product in water. The highest degree of dispersibility for the PAA-GNTs was achieved by using a 5 : 1 weight ratio of AA to US-tubes. Furthermore, the pH of the reaction had to be maintained above pH 4.5 to prevent loss of Gd^3+^ ions from the PAA-GNT product, which began to occur by pH 4.0 [39]. Under these conditions, the PAA-GNT product contained 4.5 wt.% Gd.

**Figure 2:**
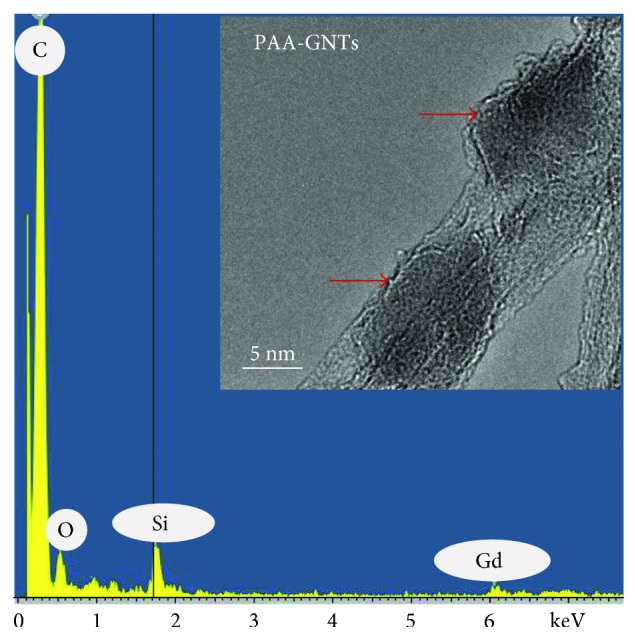
EDS data for the PAA-GNTs; inset: HRTEM image of the PAA-GNTs.

The TEM image of the PAA-GNT material (Figure 2, inset) showed that the general structure of the US-tubes was preserved after the in situ radical polymerization procedure to produce the PAA-GNTs. The darker areas of the inset of Figure 2, as indicated by the red arrows, suggest the presence of polymer on/around the GNTs. As expected, electron dispersive spectroscopy (EDS), also shown in Figure 2, demonstrated the presence of carbon, oxygen, and gadolinium for the PAA-GNT product. The silicon peak is an artifact related to the Si content of the EDS detector [40].

**Figure 3:**
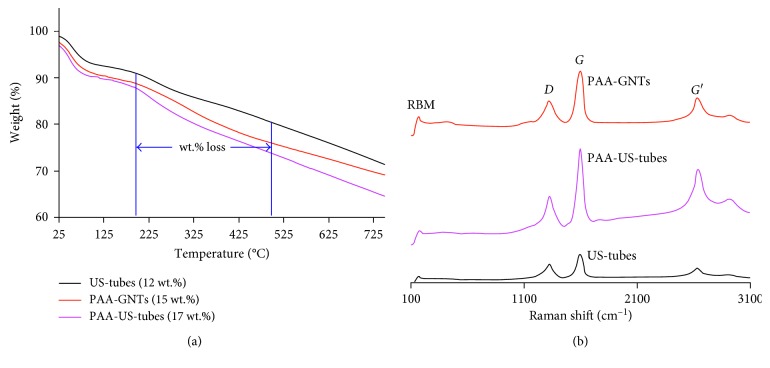
(a) TGA data and (b) Raman spectroscopy data for the US-tubes, PAA-US-tubes, and PAA-GNTs.

TGA data for the PAA-GNTs were used to confirm the presence of US-tubes wrapped with PAA polymer, as shown in Figure 3(a). TGA profiles showed greater weight loss with increasing temperature for samples with PAA content (PAA-US-tubes and PAA-GNTs) compared to the US-tubes alone. The first weight loss took place mostly in the 200–500°C range, probably because of decarboxylation either from the carboxylate groups at US-tube or GNT defect sites or from the PAA coating. Comparative TGA profiles for the US-tubes, PAA-US-tubes, and PAA-GNTs provided valuable information about the presence of PAA in the modified structures. Greater weight loss in the temperature range of 200–500°C for the PAA-US-tubes (17 wt.%) and PAA-GNTs (15 wt.%) versus only a 12 wt.% loss for US-tubes indicates the presence of additional carboxylate groups from the PAA coating for those structures. Raman spectra of the PAA-US-tubes and PAA-GNTs, shown in Figure 3(b), have the characteristic *D*, *G*, *G*′, and RBM bands of CNT materials, with the PAA-containing samples showing somewhat greater intensity for all the bands compared to the US-tubes alone.

### 3.2. Relaxivity and MRI Performance of PAA-GNTs

**Figure 4:**
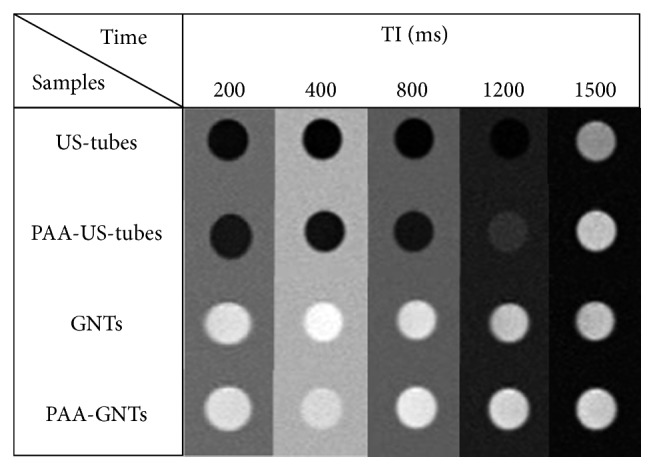
*T*_1_-weighted MR phantom images of aqueous dispersion of samples (0.9 mg/mL) acquired at 1.5 T and RT with different inversion times (TI).

To establish their properties as MRI CAs, aqueous dispersion of PAA-GNTs and PAA-US-tubes at a concentration of 0.9 mg·mL^−1^ was imaged using a 1.5 T MRI scanner. Due to poor dispersibility of US-tube and GNT samples, they were suspended in a 0.17 v/w% aqueous solution of Pluronic-108 surfactant for imaging. *T*_1_-weighted MR phantom images acquired using a 600 s inversion time (TI) demonstrated that there is clear visual contrast difference between controls with no Gd^3+^ (US-tubes and PAA-US-tubes) and the GNTs and PAA-GNTs, as shown in Figure 4.

Relaxivities for the functionalized PAA-GNTs and controls were calculated from the evolution of MR signal acquired at different inversion delays. The *r*_1_ value for the PAA-GNTs was 150 mM^−1^·s^−1^ which is comparable to the relaxivity value of GNTs reported previously [29]. This relaxivity for the PAA-GNTs in water suggests high dispersibility of the material, eliminating the need for a surfactant to achieve highly suspendable GNTs. Studies to determine the stability of Gd^3+^ within the PAA-GNTs were also performed which demonstrated that a challenge with 10% fetal bovine serum (FBS) in phosphate-buffered solution (PBS) did not produce any loss of Gd^3+^ ion after a 24/48 h challenge period (Figure 1S).

### 3.3. Cell Viability and MRI Studies of the PAA-GNT-Labeled MSCs

**Figure 5:**
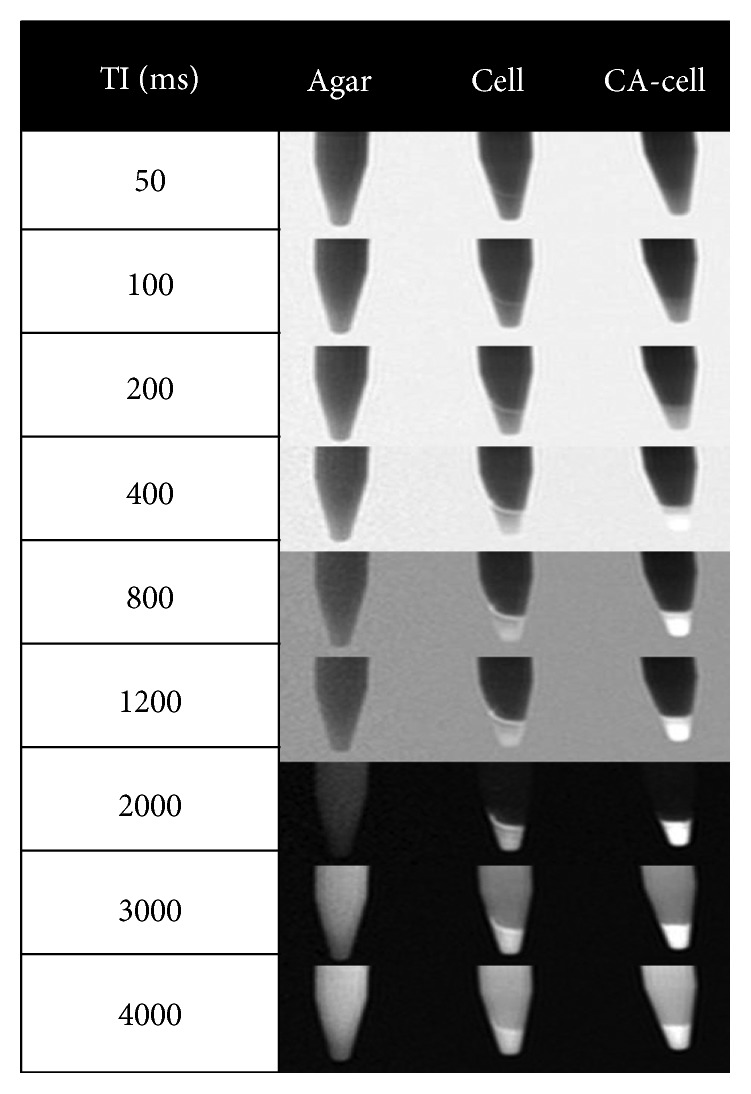
*T*_1_-weighted MR images of the samples acquired at 1.5 T and RT. Left to right: agar in water (agar), control sample of 3 × 10^7^ unlabeled MSCs (cell), and 3 × 10^7^ PAA-GNTs-labeled MSCs (CA-cell). All cell-containing samples were in a 0.5% agarose gel.

Before evaluating the performance of the PAA-GNT material as an intracellular CA (Figure 5), its cytotoxicity in MSCs was examined using FACS analysis to determine the viability of labeled cells compared to unlabeled control cells. After incubating MSCs with PAA-GNTs (20 *µ*M Gd^3+^) for 24 h, the uptake of PAA-GNTs by the cells was confirmed and quantified by ICP-OES analysis. Approximately 10^14^ Gd^3+^ ions/cell were successfully taken up, which is a significantly higher concentration of Gd^3+^ ions/cell than was previously taken up using Pluronic-wrapped GNTs (10^9^ Gd^3+^ ions/cell) [2]. Cytotoxicity studies using FACS (Figure 2S) showed no difference in viability measured by calcein staining, demonstrating that the membrane integrity of the cells was not compromised and that the MSCs remained highly viable after 24 hours of exposure to PAA-GNTs. As shown in Supplemental Figure 3S, the percentage of dead cells was 2.8% (SEM = 0.7) in control samples; meanwhile, the PAA-GNT samples showed 2.1% (SEM = 0.3). After demonstrating that PAA-GNTs can be internalized into MSCs to deliver a high concentration of Gd^3+^ ions/cell safely, MR images of the labeled MSCs were obtained. Agarose gel (0.5 mL of 0.5%) was added on the top of a pellet containing 3 × 10^7^ PAA-GNT-labeled and unlabeled MSCs. The resulting MSC pellets and a water phantom were then imaged using a 1.5 T MRI scanner. The *T*_1_-weighted MR images clearly demonstrate the rapid MR signal recovery of labeled cells compared to unlabeled cells and water phantom images (Figure 5), which is especially conspicuous in images acquired at TIs in the range of 800–3000 ms.

**Figure 6:**
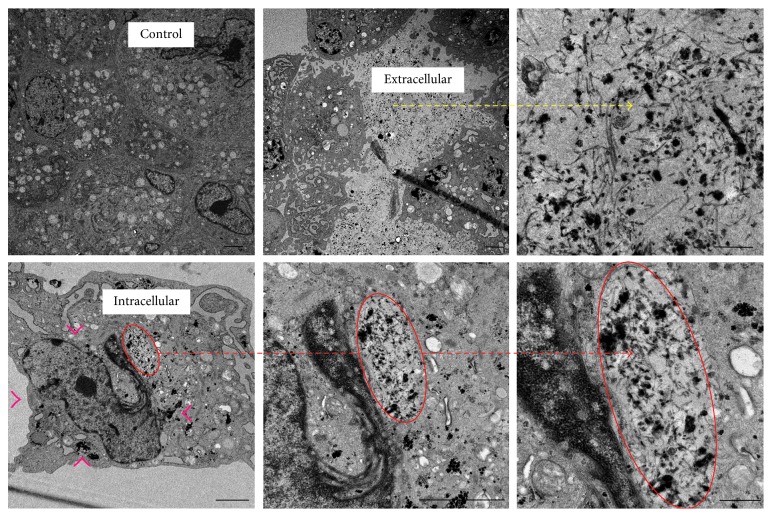
Representative TEM images of MSCs labeled with PAA-GNTs. Red circles indicate the intracellular PAA-GNTs localized in the cytoplasm of the cells, red arrowheads show scattered PAA-GNTs, and the yellow arrow shows PAA-GNTs in the extracellular space. Scale bars = 2 *µ*m.

TEM images of the PAA-GNT-labeled MSCs demonstrated that the PAA-GNTs appear as an accumulation of mostly small separated bundles of electron-dense aggregates of PAA-GNTs within the MSCs (Figure 6). In general, the material is not encapsulated within vesicles but appears to accumulate and aggregate in small clusters/bundles within the cytoplasm. From the TEM images, it is also apparent that PAA-GNTs do not enter the nucleus, which is preferable since foreign materials within the nucleus could interact and adversely alter DNA within cells.

The mostly very small bundles of PAA-GNTs found in the cytoplasm of MSCs in the present study are strikingly different from the very large bundles observed in the cytoplasm after labeling with surfactant-wrapped GNTs in our previous study [2]. We suggest that this notable difference may be due to the fact that the surfactant coating of surfactant-wrapped GNTs may be stripped off during the cell labeling process, which, in turn, encourages aggregation of the GNTs once they are internalized in the cell. Since the PAA coating of the PAA-GNTs appears to be stable in cells, it may be that there is some enhanced intermolecular interaction created between the coating and the GNTs that keeps it firmly attached when the AA polymerizes on the surface of the GNTs which might involve the GNT carboxylic acid groups at the defect sites. Thus, the PAA-GNTs appear to be a superior cell labeling agent compared to surfactant-wrapped GNTs with better dispensability in biological media which results in greater cellular uptake with 10^14^ Gd^3+^ ions/cell versus 10^9^ ion/cell for surfactant-wrapped GNTs [2].

## 4. Conclusion

In summary, this work has demonstrated that in situ surface polymerization of acrylic acid onto GNTs produces a highly water-dispersible counterpart, the PAA-GNTs, while maintaining the same relaxivity as surfactant-wrapped GNTs (150 mM^−1^·s^−1^). The PAA-GNT material can be dispersed in aqueous solution to the extent of approximately 20 mg·mL^−1^ without the use of a surfactant. Furthermore, it has been shown that the PAA-GNT CAs can be safely used to internally label porcine bone-marrow-derived MSCs to visualize the cells with MRI with potential applications for monitoring transplanted stem cells *in vivo*. Due to the enhanced stability in aqueous solution as well as in cells without the need of a surfactant, this new, highly water-dispersible PAA-GNT material appears to be a better cell labeling agent than surfactant-wrapped GNTs.

## Data Availability

The data used to support the findings of this study are available from the corresponding author upon request.

## Supplementary Materials

Supplementary Materials Supplementary information includes details on how the material was challenged for stability in biological media over time (Figure 1S), cell viability studies (Figure 2S), and average dead cell population frequency (Figure 3S) of control and material-treated MSCs.
