# Supplementary material for: A New High-Performance Gadonanotube-Polymer Hybrid Material for Stem Cell Labeling and Tracking by MRI
Source: Contrast Media Mol Imaging. 2018 Jul 10;2018:2853736. doi: 10.1155/2018/2853736 (PMC6079544; doi:10.1155/2018/2853736)
Supplement: Supplementary Materials — Supplementary information includes details on how the material was challenged for stability in biological media over time (Figure 1S), cell viability studies (Figure 2S), and average dead cell population frequency (Figure 3S) of control and material-treated MSCs. [file 2853736.f1.pdf]

# Supporting Information

## A New High-Performance Gadonanotube Polymer-hybrid Material for Stem Cell Labeling and Tracking by MRI

**Sakineh E. Moghaddam[a], Mayra Hernández-Rivera[a], Nicholas G. Zaibaq[a], Afis Ajala[b,c], Maria da Graça Cabreira-Hansen[d], Saghar Mowlazadeh-Haghighi[a] James T. Willerson[d], Emerson C. Perin[d], Raja Muthupillai[b], and Lon J. Wilson[a*]**

[a]*Department of Chemistry and The Smalley-Curl Institute, M.S. 60, Rice University, P.O. Box 1892, Houston, TX 77251-1892, USA*
[b]*Department of Radiology, Baylor St. Luke's Medical Center, 6720 Bertner Avenue, MC 2-270, Houston, TX 77030-2697, USA*
[c]*Department of Physics, University of Houston, Houston, TX 77004, USA*
[d]*Stem Cell Center, Texas Heart Institute at Baylor St. Luke's Medical Center, MC 2-255, P. O. Box 20345, Houston, TX 77225-0345, USA*

In an effort to better understand the stability of the PAA-GNTs in water, 20 mg of the material was dispersed in 10 mL water, sonicated for 30 min, and then left to stand for 5 min, 1 d, 1 w, and 2 w. Thereafter, UV-Vis spectroscopy was used to monitor PAA-GNT signal intensity with time for each sample. From these data, it was determined that the change in PAA-GNT concentration with time was essentially zero for all time points. This means that the PAA-GNT suspension was stable with time for up to 2 w. In addition, a PAA-GNT sample suspended in 10% FBS for up to 48 h resulted in no loss of $Gd^{3+}$ ion from the sample as determined by inductively-coupled plasma mass spectrometry (ICP-MS, Perkin Elmer NexION 300X) and as shown schematically in Figure 1S.

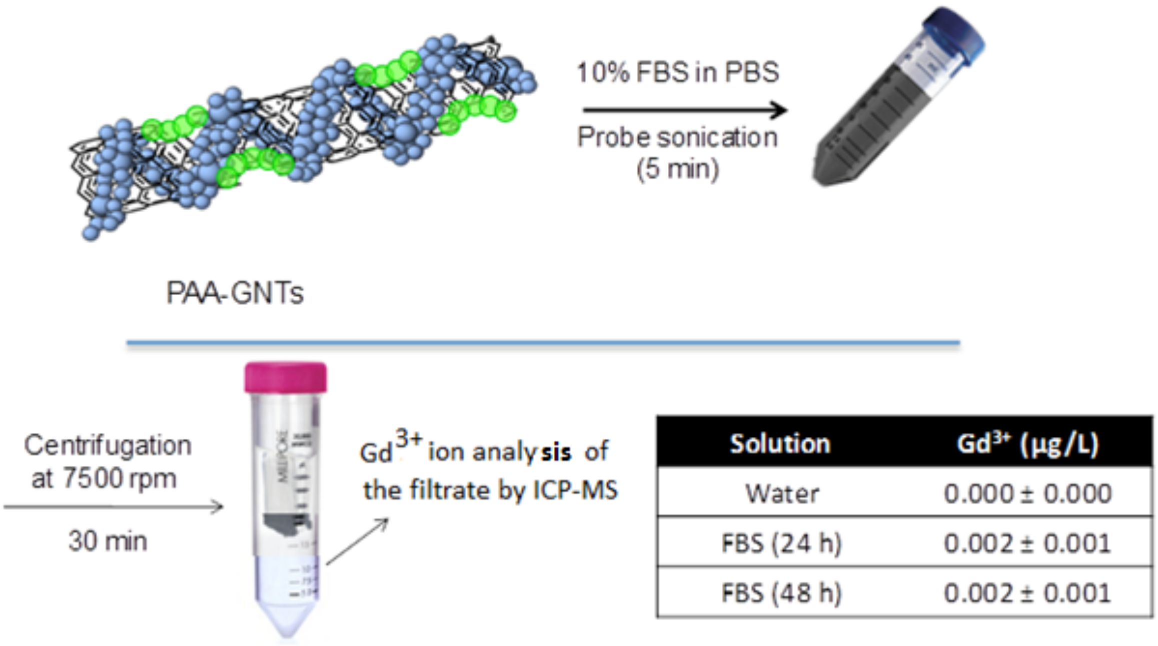


**Figure 1S.** Schematic diagram of the 10% FBS challenge experiment at 37 °C demonstrating no detectable loss of $Gd^{3+}$ ion from PAA-GNTs.

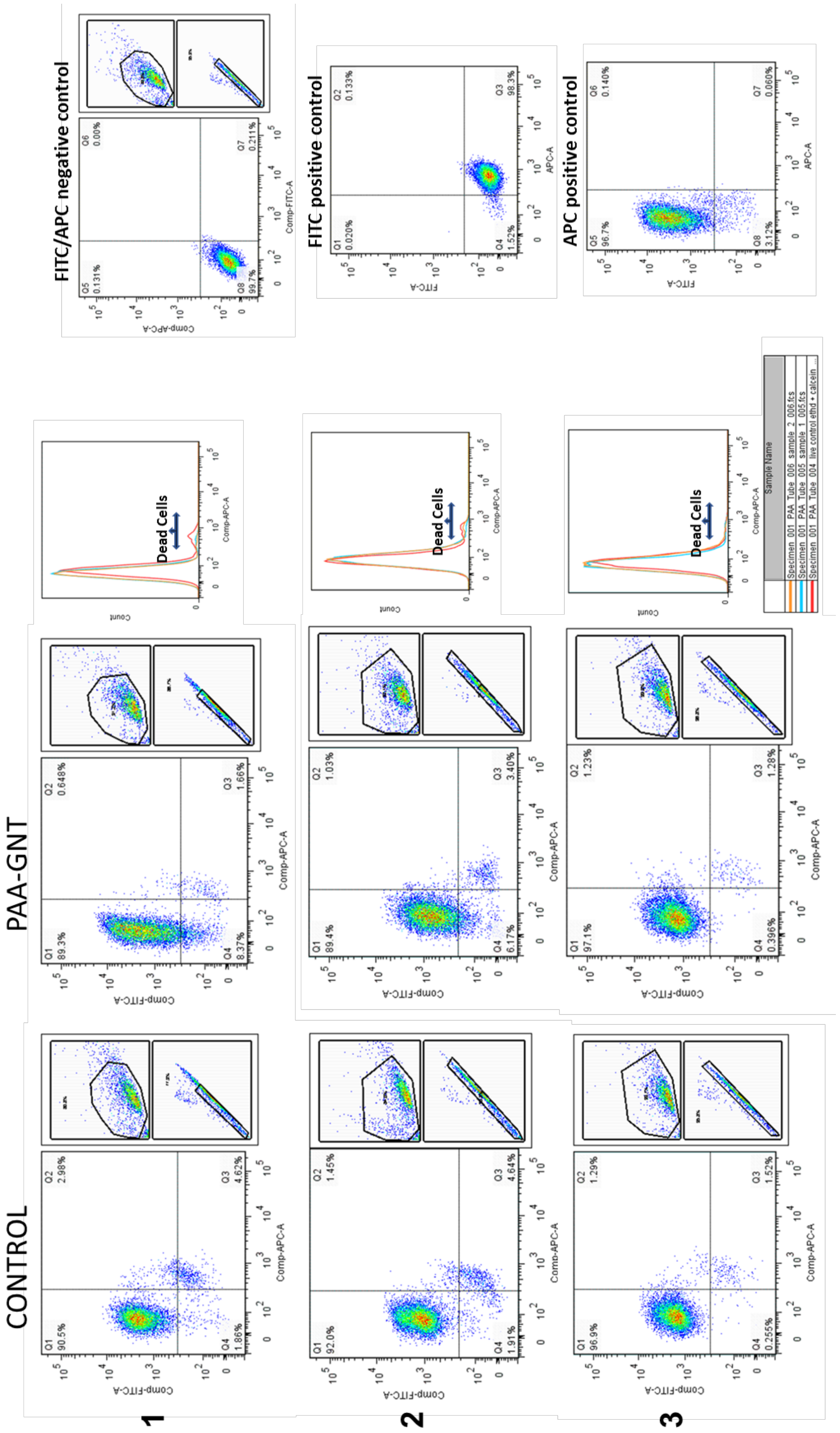
CONTROL
PAA-GNT
FITC/APC negative control
FITC positive control
APC positive control
Dead Cells
1
2
3

**Figure 2S:** Viability assay obtained from pig (N=3) bone marrow-derived MSC lines. Live cells were double stained with Calcein and detected in the green/FITC channel (y axis), meanwhile staining with Ethidium Homodimer-1 was used to determine the dead cell population detected in the red/ APC channel (x axis). Plots representative of each MSC line showing unlabeled control and one replicate from the PAA-GNT 24-hour exposure are displayed in the rows. The two additional small plots show the ancestral gates applied to compensated APC and FITC fluorescence data. To depict dead cells, a histogram of the red fluorescence is presented showing the control unlabeled (red line) cells and two PAA-GNT replicates (blue and orange). One representative plot of each control preparation is also shown on the right column. Unlabeled, no staining cell preparations were used as the universal negative control. Positive control for live cells were unlabeled cells single stained with Calcein. Dead cell positive control was prepared by treating unlabeled cells with methanol followed by single staining with Ethidium Homodimer-1.

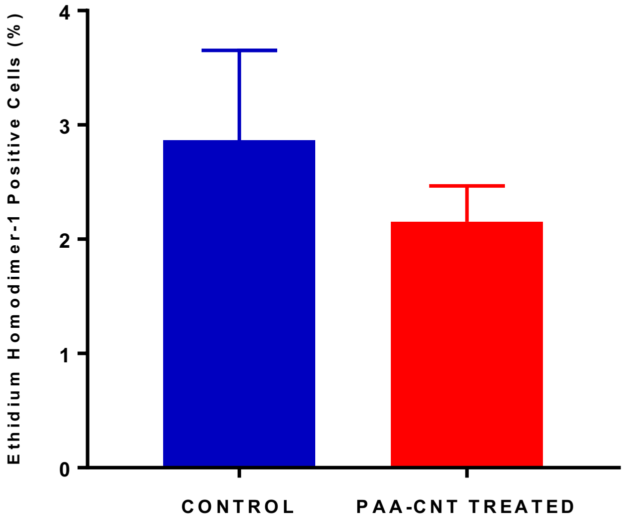


**Fig. 3S**: Average dead cell population frequency (%) in control unlabeled MSC cultures isolated from 3 pig bone marrows and in simultaneously PAA-GNT-labeled counterparts.
